# Heparin-Induced Hemorrhagic Bullous Dermatosis: A Rare Complication of Unfractionated Heparin

**DOI:** 10.7759/cureus.63676

**Published:** 2024-07-02

**Authors:** Rida Zakar, Nader Saad, Marie El Tannoury, Boutros El Tannoury, Mohammad Ali Ismail, Moussa Riachy, Lamisse Karam

**Affiliations:** 1 Medical School, Université Saint Joseph, Beirut, LBN; 2 Thoracic and Vascular Surgery, Hôtel-Dieu de France, Beirut, LBN; 3 Pulmonary and Critical Care Medicine, Hôtel-Dieu de France, Beirut, LBN; 4 Orthopaedic Surgery, Centre Hospitalier Universitaire Notre Dame Des Secours, Holy Spirit University of Kaslik, Kaslik, LBN; 5 Anesthesia and Critical Care, Hôtel-Dieu de France, Beirut, LBN

**Keywords:** pulmonary embolism (pe), deep vein thrombosis (dvt), non-fractioned heparin, heparin administration, bullous haemorrhagic dermatosis

## Abstract

We present a case of an 82-year-old female with a significant medical history of hypertension and Alzheimer’s disease who developed heparin-induced hemorrhagic bullous dermatosis during treatment for a subsegmental pulmonary embolism. The patient was admitted with lower extremity edema and cyanosis, diagnosed with a subsegmental pulmonary embolism, and started on therapeutic doses of unfractionated heparin. On the sixth day of heparin therapy, she developed abdominal bloating and a diffuse exanthematous rash, which progressed to hemorrhagic bullae on the plantar and dorsal aspects of her feet, alongside extensive purpura on her legs. Laboratory findings revealed thrombocytopenia. Multidisciplinary consultations confirmed the diagnosis of heparin-induced hemorrhagic bullous dermatosis. Management included continuing unfractionated heparin with close monitoring, supportive topical treatments, and a subsequent transition to rivaroxaban. The patient’s condition improved significantly, and she was discharged in stable condition. This case highlights the importance of recognizing rare adverse reactions to heparin and raises the question of preventive measures or risk factors related to this manifestation.

## Introduction

Heparin is a widely used anticoagulant for the prevention and treatment of thromboembolic disorders, and its adverse skin reactions, though uncommon, are significant when they occur. Heparin-induced bullous dermatosis (HIBD) is a rare dermatological condition characterized by the development of hemorrhagic bullae, typically appearing at sites distant from heparin injection locations. This condition, first described in 2006, is often under-recognized and can pose diagnostic and management challenges [1]. The exact pathophysiology of HIBD remains unclear, but it is hypothesized to involve immune-mediated processes and possibly a dose-dependent reaction [2].

Clinically, this condition presents with hemorrhagic bullae that typically appear 5-21 days after the initiation of heparin therapy [3]. These lesions are usually asymptomatic and are most commonly found on the extremities and trunk [4]. Histopathological examination of biopsy samples generally reveals intraepidermal or subepidermal hemorrhagic blisters filled with red blood cells, without significant inflammatory infiltrate [5].

The literature reports varying outcomes and management strategies for HIBD. While some studies highlight the need to discontinue heparin therapy and switch to alternative anticoagulants [6], others suggest that the condition is self-limiting and resolves within a few weeks, regardless of whether heparin therapy is continued [7]. Additionally, the risk of significant complications, such as extensive skin involvement or secondary infections, underscores the importance of early recognition and appropriate management [8].

This case report describes a patient who developed HIBD following the administration of unfractionated heparin at maximum infusion rates. We detail the clinical presentation and management approach, in order to critically examine the patient’s case and reflect on possible risk factors and preventive measures that can be taken.

## Case presentation

The patient was an 82-year-old female with a past medical history notable for hypertension and Alzheimer’s disease, managed with memantine and molsidomine. The patient was also on quetiapine 300 mg for three years for behavioral disturbances, recently tapered to 150 mg to introduce a new medication.

**Figure 1:**
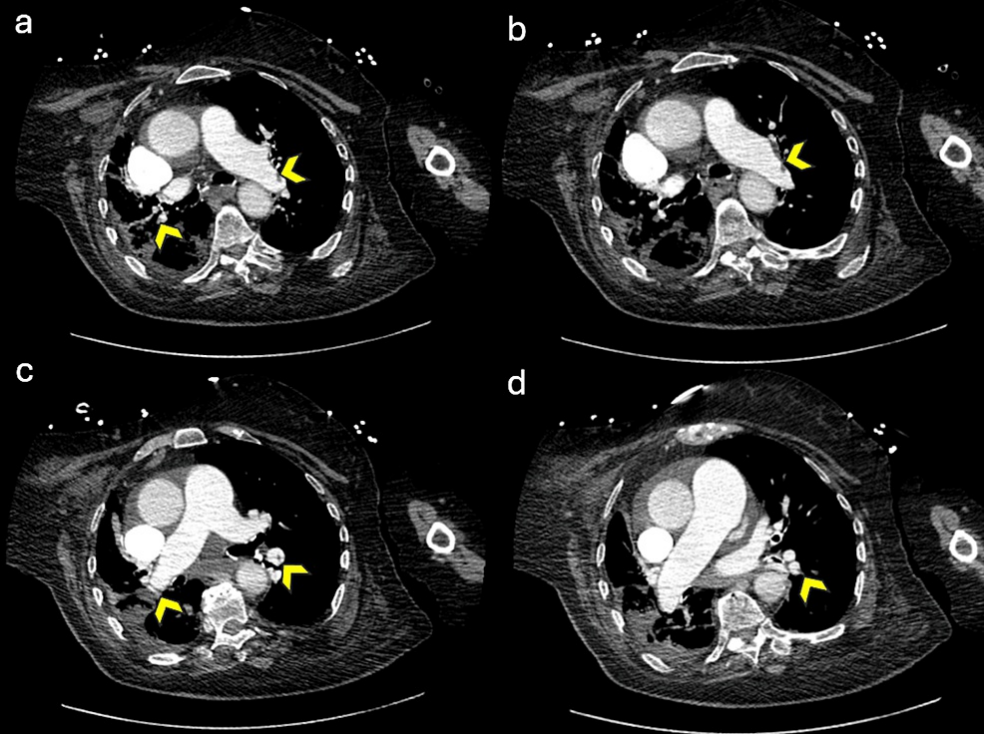
CT pulmonary angiography showing partial and multiple segmental opacification defects indicative of small pulmonary emboli A partial opacification defect is seen in the pulmonary arterial branch destined for the left lower lobe and several segmental and subsegmental opacification defects in both lung fields (arrows), particularly visible in the right upper lobe and right lower lobe, consistent with multiple small pulmonary emboli.

**Figure 2:**
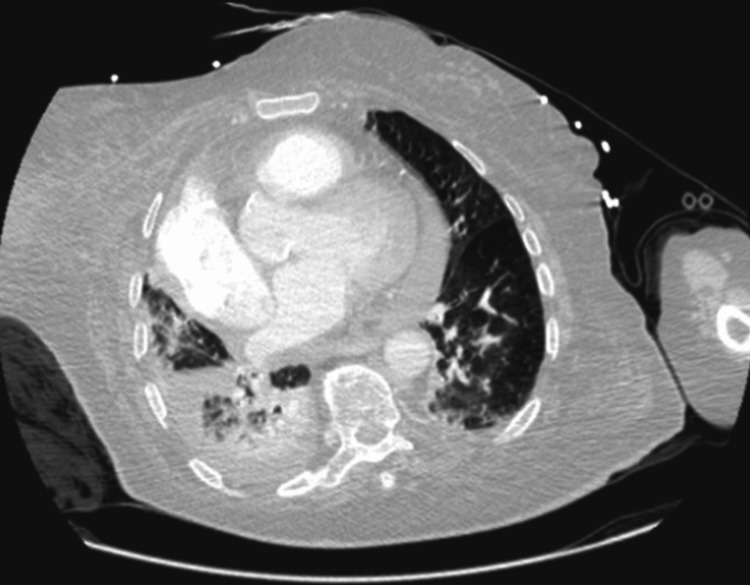
Chest CT showing alveolar and ground-glass infiltrates in both lung fields, with some located peripherally and circumscribed, suggesting atypical pneumonia or pulmonary infarctions, alveolar consolidation of the right basal pyramid, and small right pleural and pericardial effusions.

On day one of hospitalization, the patient was admitted with dyspnea and desaturation in addition to lower extremity edema and cyanosis. An initial assessment revealed a subsegmental pulmonary embolism, as the CT pulmonary angiography showed partial opacification defects in the arterial branch to the left lower lobe and multiple segmental and subsegmental opacification defects in both lung fields, particularly in the right upper and lower lobes (Figure 1). Additionally, it showed alveolar and ground-glass infiltrates in both lung fields, suggesting probable pneumonia or pulmonary infarctions (Figure 2). Laboratory results showed elevated blood urea nitrogen (22.2 mg/dL), normal creatinine (0.75 mg/dL), elevated sodium (153 mmol/L), normal potassium (3.9 mmol/L), hemoglobin of 13.7 g/dL, white blood cell count of 16,000/mm³ with 85% neutrophils, and a platelet count of 100,000/mm³ and a C-reactive protein of 63 mg/L. Liver function tests were very slightly elevated and remained unchanged throughout the patient’s hospital stay.

Therapeutic doses of unfractionated heparin (up to 35,000 units) were then initiated on the spot as well as empirical antibiotherapy by piperacillin-tazobactam and oxygen therapy. A subsequent lower limbs ultrasonography revealed a deep vein thrombosis involving the right calf veins and extending to the popliteal vein, with the head of the thrombus stopping at the supragenicular popliteal vein.

**Figure 3:**
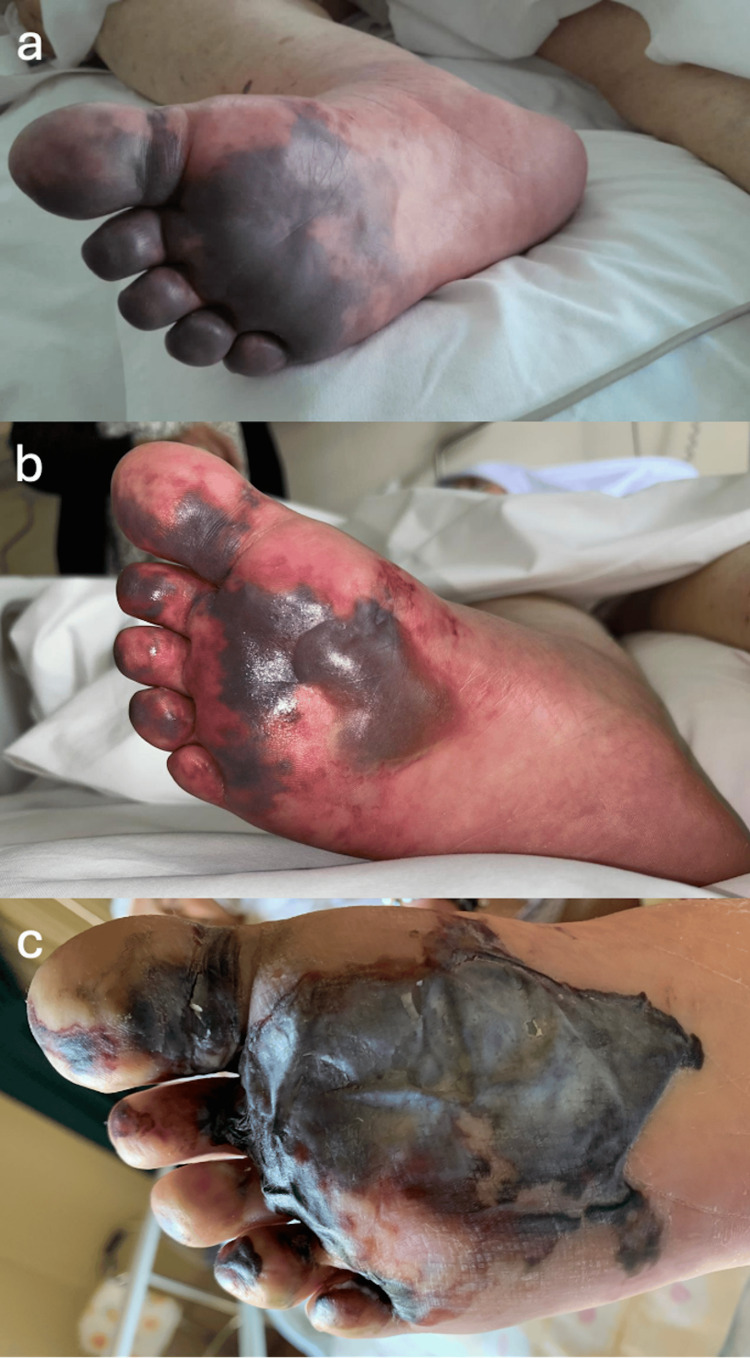
Evolution of the apparent hemorrhagic bullous dermatosis on the plantar foot. (a) First appearance of extensive purpuric lesions on right forefoot, on day 4 after heparin initiation; (b) Fully developped bullous hemorrhagic lesion on right forefoot, on day 6 after heparin initiation; (c) Aspect of right foot on the day of discharge, on day 14 after first lesion appearance.

**Figure 4:**
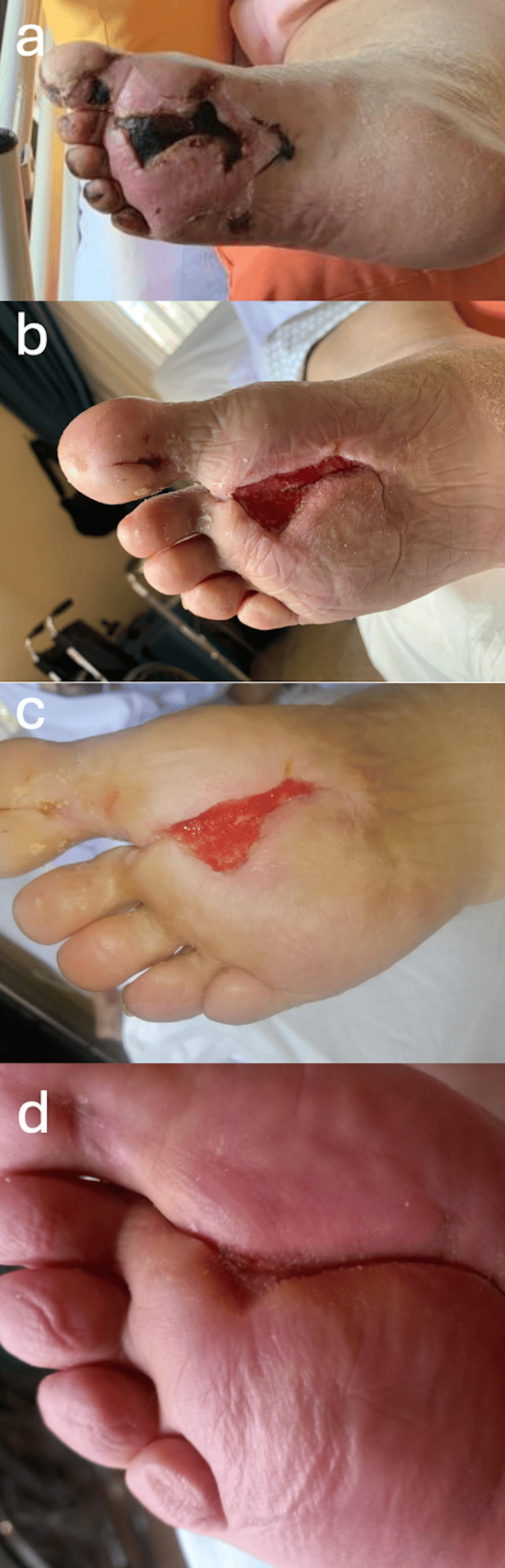
Evolution of the plantar lesion (a) one week after discharge, (b) following debridement, (c) two months after discharge, and (d) three months after discharge; improvement with negative pressure therapy.

**Figure 5:**
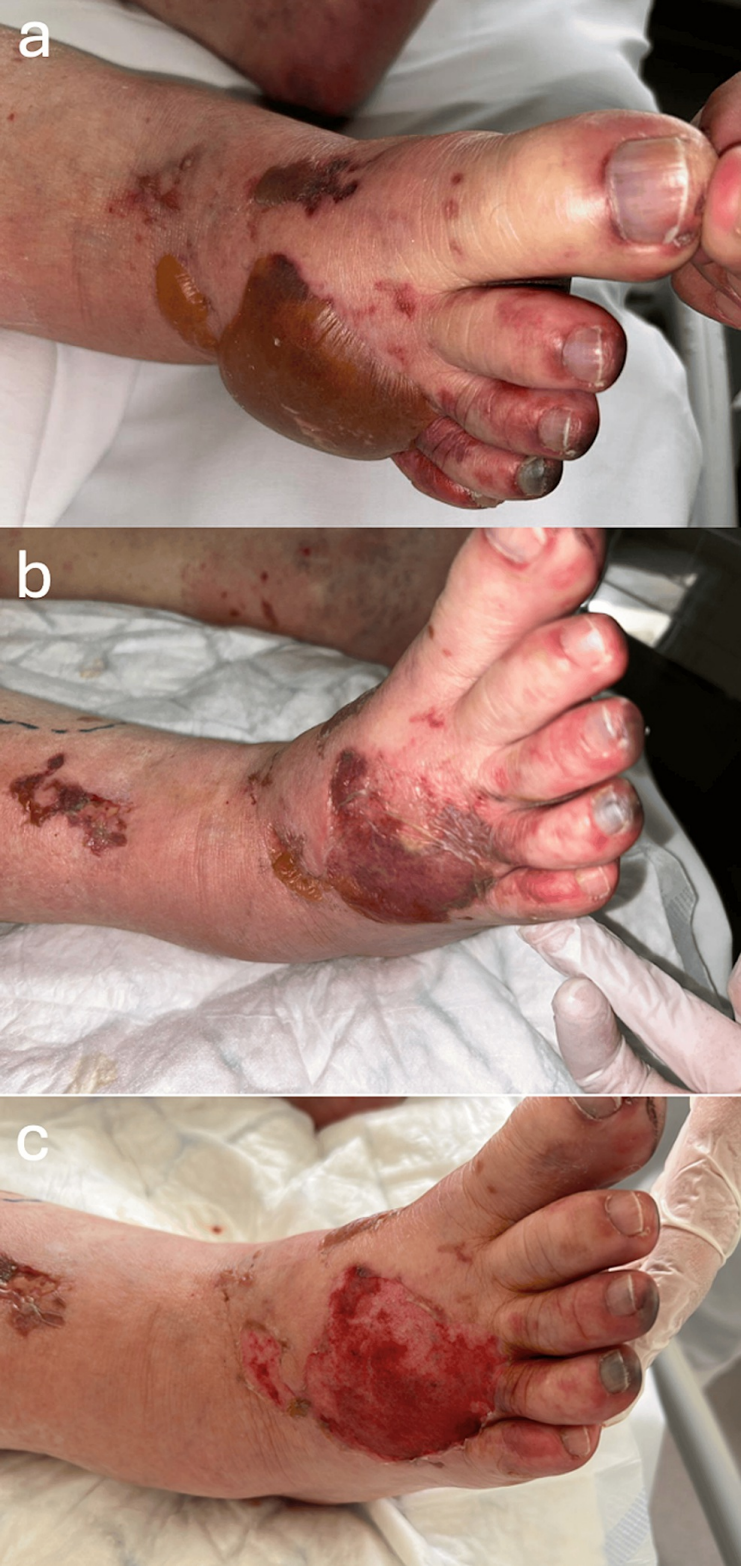
Chronological evolution of an apparent complex lesion, with bullae on the dorsal aspect of the foot along the extensive purpura. Note: The blister does not appear to be entirely hemorrhagic, but could be related to the patient's exhibited HIBD. HIBD: heparin-induced bullous dermatosis

**Figure 6:**
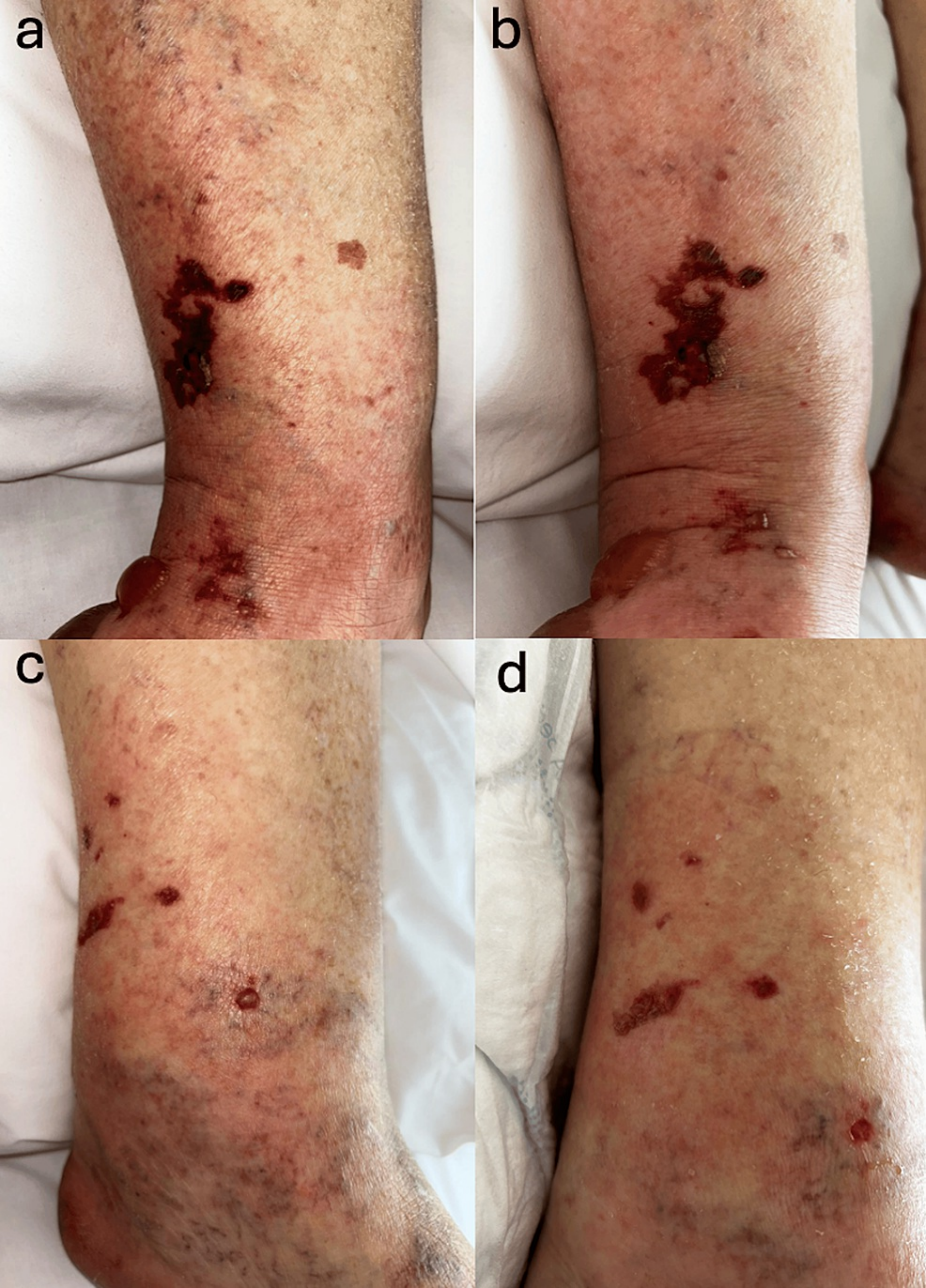
Changes in two distinct lesions, with visible hemorrhagic bullae evolving into crusting and desquamating skin after topical treatment. (a-b) Pretibial complex bullous hemorrhagic lesion evolving throughout the patient's hospital stay; (c-d) A posterior tibial hemorrhagic blister (with nearby lesions) evolving throughout the patient's hospital stay.

On day four, the patient developped extensive purpura on the plantar and dorsal aspects of her right foot. Within two days, serous bullae became evident and quickly evolved into hemorrhagic bullae. Meanwhile, smaller hemorrhagic blisters were also noted at the distal third of the leg. The evolution of these lesions can be visualized in Figures 3-6.

Later on that day, the patient developed a diffuse exanthematous rash on her back. No improvement was noted with the use of oral antihistamines (desloratadine and bilastine). Empirical antibiotic therapy was also suspended at this point and was resumed later on.

As the patient displayed a complex constellation of symptoms, a multidisciplinary approach was adopted. A diagnosis of syndrome of inappropriate antidiuretic hormone secretion (SIADH) was made by the internist, explaining the aforementioned polyuria, and the diagnosis of HIBD was made by the vascular surgeon. It is worth noting that the platelet count had dropped to 82,000/mm³ that day.

Recommendations included continuing unfractionated heparin under close monitoring of the skin lesions and platelet count while applying protective dressings. Supportive measures for the back rash included topical corticosteroids (clobetasol 0.05%) once daily for 15 days. The patient's pneumonia was treated successfully with piperacillin-tazobactam, with subsequent improvement in clinical and biological markers. The patient followed a total course of eight days of piperacillin-tazobactam at a dose of 4.5 grams every six hours, with a one-day stop to exclude a potential adverse reaction.

Unfractionated heparin (up to 35,000 units) was kept until day 12 when the patient was discharged under rivaroxaban 20 mg daily. Marked resolution of the skin lesions was noted two weeks later, except for the deeper lesions at the plantar forefoot that needed surgical debridement. The evolution was favorable with negative pressure wound therapy. Figure 4 shows the evolution of the lesions over time.

## Discussion

In the literature, HIBD is usually reported within 5-21 days after the initiation of heparin therapy [9]. It typically presents with hemorrhagic bullae that appear at distant locations from heparin injections. The patient in this case was under continuous intravenous therapeutic heparin therapy. She developed extensive purpura on her right leg on day 4, which progressed into hemorrhagic bullae within two days. This rapid progression is notable, as the literature often describes a more gradual onset, typically ranging from eight to 20 days after the start of heparin therapy [9]. Additionally, while many cases involve asymptomatic lesions, this patient's bullae were accompanied by systemic symptoms highlighting a more severe systemic response in the context of multiple complex pathologies.

A particularly striking aspect of this case is the extensive nature of the lesions compared to those reported in the literature. Most documented cases of HIBD describe lesions localized primarily to the extremities and trunk [2]. In contrast, this patient exhibited extensive purpura that quickly progressed into hemorrhagic bullae, covering both the plantar and dorsal aspects of her feet and extending to her legs. The rapid and widespread dissemination of these lesions underscores a more severe manifestation of HIBD, which is relatively uncommon and highlights the variability in clinical presentation [9].

Commonly reported risk factors for HIBD include advanced age, male gender, underlying chronic conditions such as cardiovascular diseases and malignancies, and prior cutaneous reactions to heparin [4]. The patient in this case, although female, aligns with the risk factor of advanced age and had underlying chronic conditions (hypertension and Alzheimer’s disease). The presence of thrombocytopenia (platelet count dropping to 82,000/mm³) further supports the association of heparin-related complications, as thrombocytopenia is a recognized risk factor for bleeding complications [6].

The pathophysiology of HIBD is not fully understood but several hypotheses have been proposed. It is believed to involve immune-mediated mechanisms, possibly related to antibodies against heparin-platelet factor 4 complexes, similar to heparin-induced thrombocytopenia [10]. The rapid onset of symptoms following heparin administration suggests a potential hypersensitivity reaction, which is consistent with other reported cases.

Interestingly, the possibility of drug interactions contributing to the non-classical systemic symptoms observed in this case warrants consideration. The patient was on multiple medications, including quetiapine, memantine, molsidomine, Daflon, and Tanakan. Daflon, a micronized purified flavonoid fraction, is often used to treat chronic venous insufficiency and hemorrhoidal disease. Tanakan, derived from *Ginkgo biloba*, is used to improve cognitive function and treat peripheral vascular diseases. While these medications are not typically associated with significant drug interactions, the combination of heparin with multiple other drugs could potentially exacerbate adverse reactions. Adverse drug reactions involving heparin, especially in the context of multiple drug use, can lead to complex interactions and exacerbate systemic symptoms. This is particularly relevant in the case of heparin-induced thrombocytopenia (HIT), where polypharmacy can increase the risk of such a reaction [11]. In this patient's case, there were no other questionable medications on the patient's list that are known to interact with heparin. This, however, highlights the need for careful review of the patient's medication regimen when diagnosing and managing HIBD.

Additionally, the use of antibiotics such as piperacillin-tazobactam has been associated with bullous dermatoses in some cases. Piperacillin-tazobactam can induce linear IgA bullous dermatosis, an autoimmune blistering disease [12,13]. Though this differs from HIBD, the potential for antibiotics to contribute to adverse dermatological reactions should be considered in the differential diagnosis.

Management of HIBD generally involves the continuation or adjustment of anticoagulant therapy, supportive skin care, and close monitoring [9]. In this case, the patient was initially managed with unfractionated heparin and later transitioned to rivaroxaban for ongoing anticoagulation. Notably, the decision to continue anticoagulation therapy despite the development of HIBD reflects current practices, as many reported cases resolved with continued heparin use or after switching to another anticoagulant [2].

The patient’s condition improved significantly with the continuation of anticoagulant therapy and supportive skin care measures, ultimately leading to the stabilization of her respiratory status and the resolution of skin lesions. Some clinicians argue that continuing heparin in the presence of such severe dermatological reactions might pose additional risks and recommend switching to alternative anticoagulants earlier [7]. However, this approach can be controversial. As the patient exhibited several concomitant medical conditions in addition to her pulmonary embolism, the decision to continue heparin therapy under close monitoring was made. This attitude aligns with the literature, which suggests that HIBD is generally self-limiting and resolves within a few weeks, regardless of whether heparin therapy is continued or switched [3].

## Conclusions

This case underscores the importance of recognizing HIBD as a potential complication of heparin therapy, particularly in elderly patients with chronic conditions. It highlights the need for close monitoring, prompt diagnosis, and appropriate management to ensure favorable outcomes. The comparison with current literature reaffirms the typical presentation, risk factors, and management strategies for HIBD while emphasizing that it can occur across a broader demographic and extent of lesions than predominantly reported.
